# Supplementary material for: Household-level effects of seasonal malaria chemoprevention in the Gambia
Source: Commun Med (Lond). 2024 May 22;4:97. doi: 10.1038/s43856-024-00503-0 (PMC11111771; doi:10.1038/s43856-024-00503-0)
Supplement: Supplementary file 2 — Supplementary file [file 43856_2024_503_MOESM2_ESM.pdf]

Supplementary File

Household-level effects of seasonal malaria chemoprevention in the Gambia

Soremekun S et al

Supplementary captions

**Name**	**Title**
**Tables**	
STable 1	Entomological baseline study
STable 2	Temperature and rainfall data from Basse Meteorological Station 2019-2021
STable 3	Akaike and Bayesian Information Criteria (AIC and BIC) for SMC coverage models by low-group cut-off value
STable 4	Prevalence and risk ratios for asymptomatic infections in 684 children aged 0-9 years, by individual number of SMC rounds received.
STable 5	Timing of SMC: Percentage and number of children who received SMC during each monthly round, by the total number of SMC rounds received.
STable 6	Incidence and hazard ratios for clinical malaria in 825 children aged 0-9 years stratified by SMC coverage in all children 0-9 years in the same household
STable 7	Asymptomatic infection prevalence in 684 of 825 children 0-9 years visited in a late season survey 27th Sept to 27th November by household level of SMC coverage in all children 0-9 years in the same household
**Figures**	
Supplementary Figure 1	Kaplan Meyer plots for clinical malaria events in **a)** all children aged 0-9 years; **b)** children aged 0-4 years; **c)** children aged 5-9 years by number of SMC rounds received
Supplementary Figure 2	Geolocations of 129 study households by community. Marker colour indicates SMC status in children in household
Supplementary Figure 3	P-values from Global Moran I tests for spatial autocorrelation in household-level regression models of malaria incidence and prevalence in participants aged 0-9yrs or 10+yrs by household coverage of SMC.

STable 1. Entomological baseline study

Entomological collections were conducted over the period survey July 2021-December 2021 in 10 communities in the Upper River Region of the Gambia. Each month over this period nightly catches took place over three days in a random selection of 6 households in each village, using Centre for Disease Control (CDC) light traps.

**Trap location**	**Mean nightly biting rate (standard deviation)***	**Min-max**	**Total trap-nights**
CDC light trap	0.97 (1.41)	0-4.94	829

**Anopheles gambiae s.l.* and *Anopheles funestus* combined.

STable 2. Maximum temperature and mean rainfall data from Basse Meteorological Station 2019-2021

**Month and year**	**Maximum temperatures (°C) per month**	**Mean rainfall (mm) per month**
**2019 malaria transmission season**		
July 2019	33.3	86.7
August 2019	32.8	343.8
September 2019	32.1	357
October 2019	33.8	76.9
November 2019	36.4	0
December 2019	36.4	0
January 2020	33.5	0
**2020 malaria transmission season**		
July 2020	32.9	314.5
August 2020	31.0	211.2
September 2020	30.8	565.7
October 2020	35.1	95
November 2020	35.6	0
December 2020	36.7	0
January 2021	37.2	0
**2021 malaria transmission season**		
July 2021	36.5	130.5

August 2021	32.9	271.4
September 2021	33.6	212
October 2021	35.2	99.4
November 2021	34.5	0
December 2021	35.2	0

STable 3. Akaike and Bayesian Information Criteria (AIC and BIC) for SMC coverage models by low-group cut-off value for i) the percentage of all children aged 0-9 years in a household who received one or more rounds of SMC and ii) the mean number of SMC rounds per child.

i

Group cut-offs for % children at least 1 round SMC (low; medium; high coverage)	Ages 10 + years		Ages 0-9 years		Number of households low; medium; high coverage groups
	AIC	BIC	AIC	BIC	
**0-24%;** 25-79%; 80%+	1285.74	1358.40	745.11	813.02	13; 59; 57
**0-29%;** 30-79%; 80%+	1285.74	1358.40	745.32	813.14	14; 58; 57
**0-34%;** 35-79%; 80%+	1287.94	1360.59	745.38	813.17	18; 54; 57
**0-39%;** 40-79%; 80%+	1288.88	1361.53	745.72	813.51	19; 53; 57
**0-44%;** 45-79%; 80%+	1290.40	1363.05	747.07	814.86	23; 49; 57
**0-49%;** 50-79%; 80%+	1290.94	1363.60	747.06	814.85	24; 48; 57
**0-54%;** 55-79%; 80%+	1289.81	1362.46	747.05	814.84	34; 38; 57

ii

Group cut-offs for mean number SMC rounds/child (low; medium; high coverage)	Ages 10 + years		Ages 0-9 years		Number of households low; medium; high coverage groups
	AIC	BIC	AIC	BIC	
**<0.3;** 0.3-2; >2	1285.72	1358.37	745.54	813.42	13; 62; 54
**<0.4;** 0.4-2; >2	1286.13	1358.78	745.65	813.70	14; 61; 54
**<0.5;** 0.5-2; >2	1287.15	1359.80	745.98	813.76	15; 60; 54
**<0.6;** 0.6-2; >2	1287.55	1360.01	745.88	813.66	20; 55; 54
**<0.7;** 0.7-2; >2	1287.78	1360.44	746.12	813.91	23; 52; 54
**<0.8;** 0.8-2; >2	1287.78	1360.44	746.12	813.91	23; 52; 54
**<0.9;** 0.9-2; >2	1288.12	1360.77	746.04	813.83	25; 50; 54

AICs and BICs are calculated following multilevel Cox models of the association between household level SMC coverage and clinical malaria in older non-recipients (ages 10+ years) and eligible children (ages 0-9 years). Models account for multiple clinical malaria episodes per person. Models adjust for village ID, ratio of children 0-9:10+ yrs, average household bed net usage over 2021 malaria transmission season and total number of household residents. Models with low-group cutoffs above 0 and less than 25% (table i) or less than 0.3 (table ii) are identical in household distribution and IC scores and are therefore not shown. Final cut-offs were chosen based on lowest IC values (25% and 0.3).

Supplementary Figure 1 Kaplan Meyer plots for clinical malaria events in **a)** all children aged 0-9 years; **b)** children aged 0-4 years; **c)** children aged 5-9 years by number of SMC rounds received during the 2021 malaria transmission season in the Upper River Region the Gambia. Grey vertical lines indicate the first reported day of each monthly rou

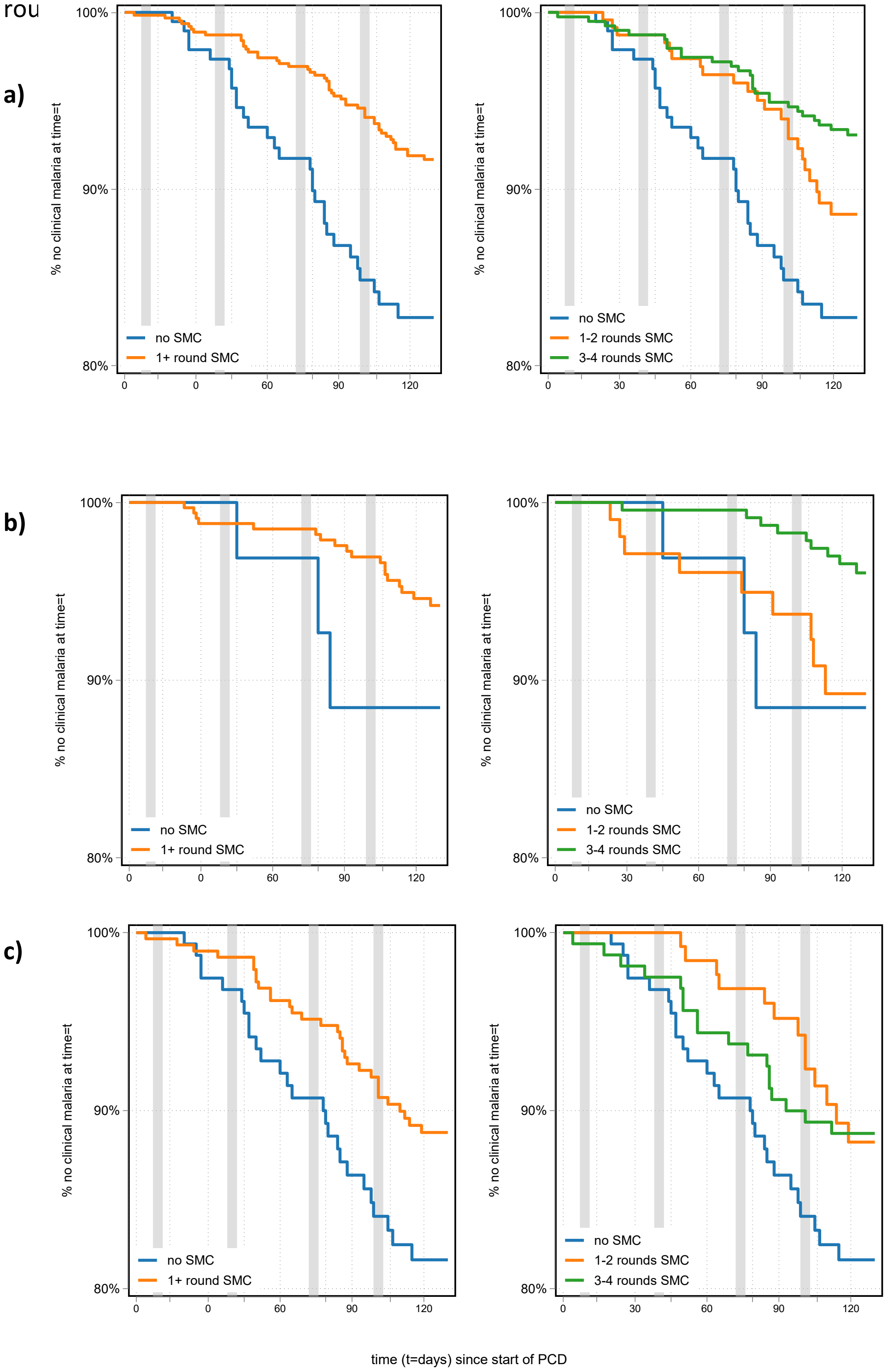

Left hand column: SMC coverage as binary categorisation (no SMC/any SMC). Right hand column: No SMC, Any SMC category further divided into 1-2 rounds and 3-4 rounds of SMC

STable 4. Prevalence and risk ratios for asymptomatic infections in 684 children aged 0-9 years, by individual number of SMC rounds received. Children were screened during cross sectional surveys conducted late in the peak malaria transmission season (27th September-27th November 2021).

**Individual SMC Coverage**	**Prevalence asymptomatic infection n/N (%)**	**Unadjusted**		**Fully adjusted**		**Prevalence high density infection n/N (%)***
		**RR (95% Ci) p**	**RR (95% Ci) p**	**RR (95% Ci) p**	**RR (95% Ci) p**	
**No SMC**	6/136 (4.4%)	comparator		comparator		2/6 (33.3%)
**Any SMC**	22/548 (4.0%)	**0.91** (0.38-2.20) 0.834		**1.74** (0.68-4.46) 0.248		10/22 (45.5%)
1-2 rounds	13/201 (6.5%)	**1.47** (0.57-3.76) 0.426	comparator	**2.05** (0.78-5.37) 0.146	comparator	6/13 (46.2%)
3-4 rounds	9/347 (2.6%)	**0.59** (0.21-1.62) 0.304	**0.40** (0.17-0.92) 0.031	**1.31** (0.42-4.14) 0.642	**0.64** (0.26-1.60) 0.340	4/9 (44.4%)

n/N is the total children with an asymptomatic infection out of the total children visited during the cross sectional survey. Fully adjusted models included village ID and the % of nights during the season the child used an insecticide treated net the night before when asked. * Due to small cell sizes models assessing SMC impact on risk of high density infections have been omitted.

STable 5. Timing of SMC: Percentage and number of children who received SMC during each monthly round, by the total number of SMC rounds received.

age group	total rounds SMC received	% (and number) of children received SMC at each monthly round				
		August	September	October	November	N
age 0-4yrs	0	0% (0)	0% (0)	0% (0)	0% (0)	0
age 5-9yrs	0	0% (0)	0% (0)	0% (0)	0% (0)	0
age 0-4yrs	1	37.2% (16)	41.9% (18)	11.6% (5)	9.3% (4)	43
age 5-9yrs	1	23.1% (18)	39.7% (31)	23.1% (18)	14.1% (11)	78
age 0-4yrs	2	34% (36)	23.6% (25)	27.4% (29)	15.1% (16)	106
age 5-9yrs	2	21.4% (21)	30.6% (30)	31.6% (31)	16.3% (16)	98
age 0-4yrs	3	28.4% (98)	30.7% (106)	19.4% (67)	21.4% (74)	345
age 5-9yrs	3	21.6% (68)	31.4% (99)	28.6% (90)	18.4% (58)	315
age 0-4yrs	4	25% (110)	25% (110)	25% (110)	25% (110)	440
age 5-9yrs	4	25% (54)	25% (54)	25% (54)	25% (54)	216

STable 6. Incidence and hazard ratios for clinical malaria in 825 children aged 0-9 years stratified by SMC coverage in all children 0-9 years in the same household over the 2021 malaria transmission season in the Upper River Region of The Gambia.

SMC coverage	**Incidence rates per 100 person-months (cases/PM)**		Unadjusted **HR (95% CI) p**		Fully adjusted **HR (95% CI) p**	
	0-9years: all	0-9years: no SMC*	0-9years: all	0-9years: no SMC	0-9years: all	0-9years: no SMC
**% Children in household received at least 1 round SMC**						
25%	**4.26** (11/259)	**5.00** (11/220)	1 (ref)	1 (ref)	1 (ref)	1 (ref)
25%-75%	**2.12** (46/2166)	**2.33** (17/729)	**0.41** (0.21, 0.81) 0.010	**0.45** (0.21, 0.96) 0.038	**1.25** (0.51, 3.08) 0.620	**0.70** (0.21, 2.34) 0.558
80%	**1.35** (28/2070)	**2.40** (3/125)	**0.25** (0.12, 0.51) <0.001	**0.29** (0.06, 1.31) 0.107	**0.91** (0.35, 2.33) 0.844	**0.89** (0.11, 7.04) 0.913
**SMC Rounds/child in household**						
<0.3	**4.23** (11/259)	**5.00** (11/220)	1 (ref)	1 (ref)	1 (ref)	1 (ref)
0.3 - <2	**2.39** (48/2006)	**3.16** (20/634)	**0.46** (0.24, 0.91) 0.026	**0.57** (0.28, 1.21) 0.145	**1.40** (0.57, 3.39) 0.460	**0.98** (0.29, 3.31) 0.979
2-4	**1.17** (26/2231)	**0.00** (0/220)	**0.21** (0.10, 0.44) <0.001	**0.03x10-^{14}** p<0.001	**0.86** (0.34, 2.20) 0.756	**0.05x10^{-14}** p<0.001

Adjusted models included village ID, household size, ratio of children to adults, age in years, household ID, baseline (dry season) infection prevalence in household, % of nights household used insecticide treated nets and individual number of SMC rounds. Columns "Aged 0-9 years: no SMC" restricts analysis to children who personally received no SMC.

STable 7. Asymptomatic infection prevalence in 684 of 825 children 0-9 years visited in a late season survey 27th Sept to 27th November by household level of SMC coverage in all children 0-9 years in the same household during the 2021 malaria transmission season in the Upper River Region of The Gambia.

SMC coverage	**Prevalence of asymptomatic infection n/N (%)**	**Prevalence of high density asymptomatic infection n/N (%)**	Risk of asymptomatic infection*	
			Unadjusted **RR (95% CI) p**	Adjusted **RR (95% CI) p**
**% Children in household received at least 1 round SMC**				
25%	1/38 (2.6%)	0/1 (0.0%)	1 (ref)	1 (ref)
25%-75%	19/313 (6.1%)	8/19 (42.1%)	0.85 (0.47, 1.52) 0.584	0.70 (0.34, 1.47) 0.344
80%	8/333 (2.4%)	4/8 (50.0%)	0.67 (0.37, 1.19) 0.169	0.95 (0.44, 2.07) 0.900
**SMC Rounds/child in household**				
<0.3	1/38 (2.6%)	0/1 (0.0%)	1 (ref)	1 (ref)
0.3 - <2	18/286 (6.3%)	8/18 (44.4%)	0.93 (0.52, 1.65) 0.807	0.78 (0.37, 1.65) 0.509
>2-4	9/360 (2.5%)	4/9 (44.4%)	0.60 (0.34, 1.05) 0.071	0.87 (0.40, 1.87) 0.709

Regression models of asymptomatic infection prevalence against SMC coverage both at household-level in children 0-9 years were used where multilevel models with household random effects failed to converge. Due to small cell sizes models assessing household SMC impact on risk of high density infections have been omitted. Adjusted models included village ID, household size, age in years, household ID, week of visit, baseline (dry season) infection prevalence in household, % of nights household used insecticide treated nets, and individual number of SMC rounds.

Supplementary Figure 2: Geolocations of 129 study households by community. Marker colour indicates SMC status in children in household; marker size indicates the mean incidence of clinical malaria or prevalence of asymptomatic infection in children 0-9 years or participants 10+ years (ineligible for SMC) in household over the 2021 transmission season in the Upper River Region of The Gambia. Map axes: Y axis = latitude; X axis = longitude. Missing compounds Fula Mori Bochi=2; Bolibana=0; Madina SS=4; Tabajang=3; Njayel=6 Banni Kunda/Temanto=1; S Demba DardoS /Biram=0; Njum Bakary=2

a) Incidence of **clinical malaria** in participants **10+ years of age** by the percentage of children in household who received one or more rounds of SMC.

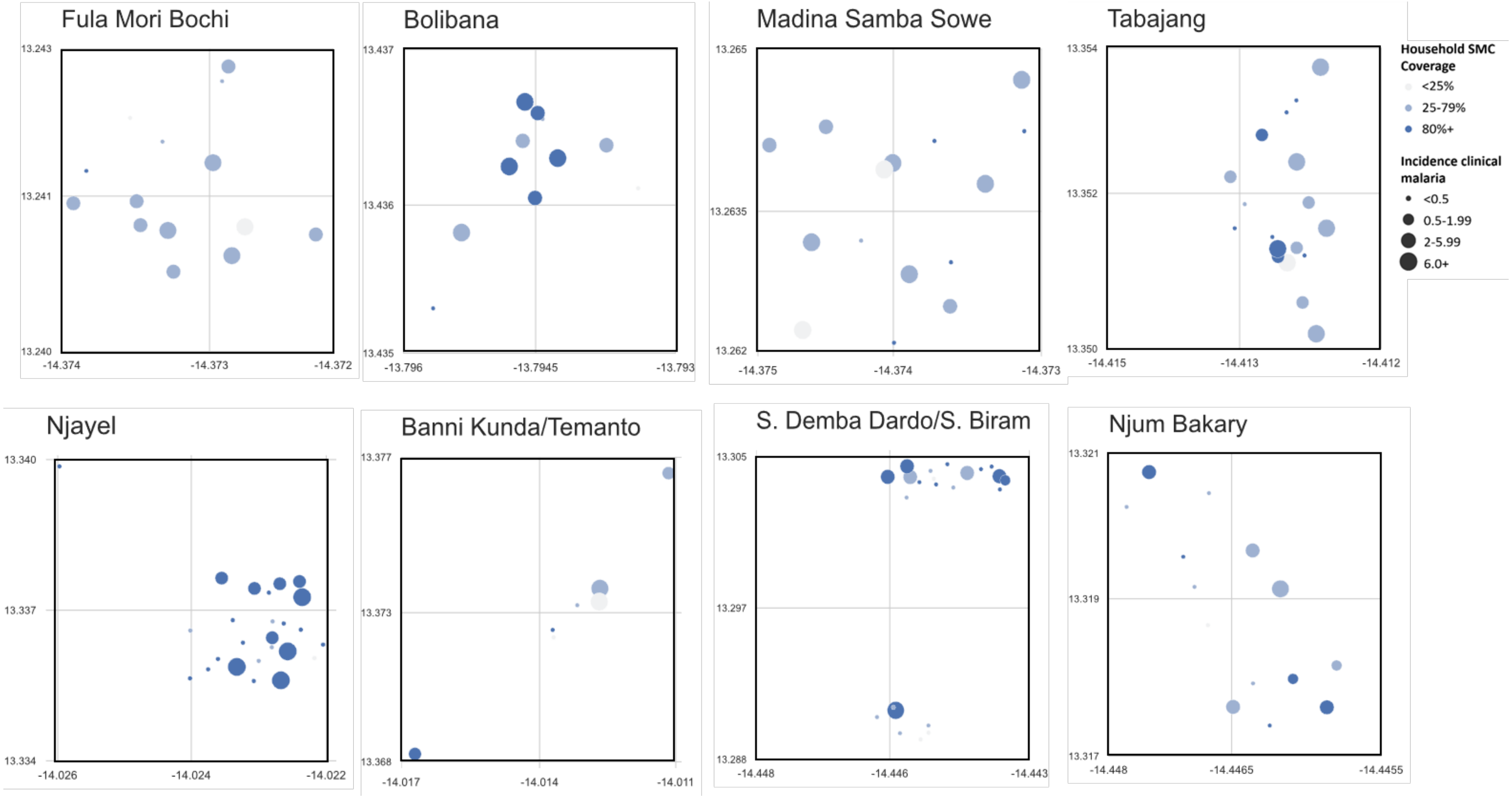

b) Incidence of **clinical malaria** in children **0-9 years of age** by the percentage of children in household who received one or more rounds of SMC.

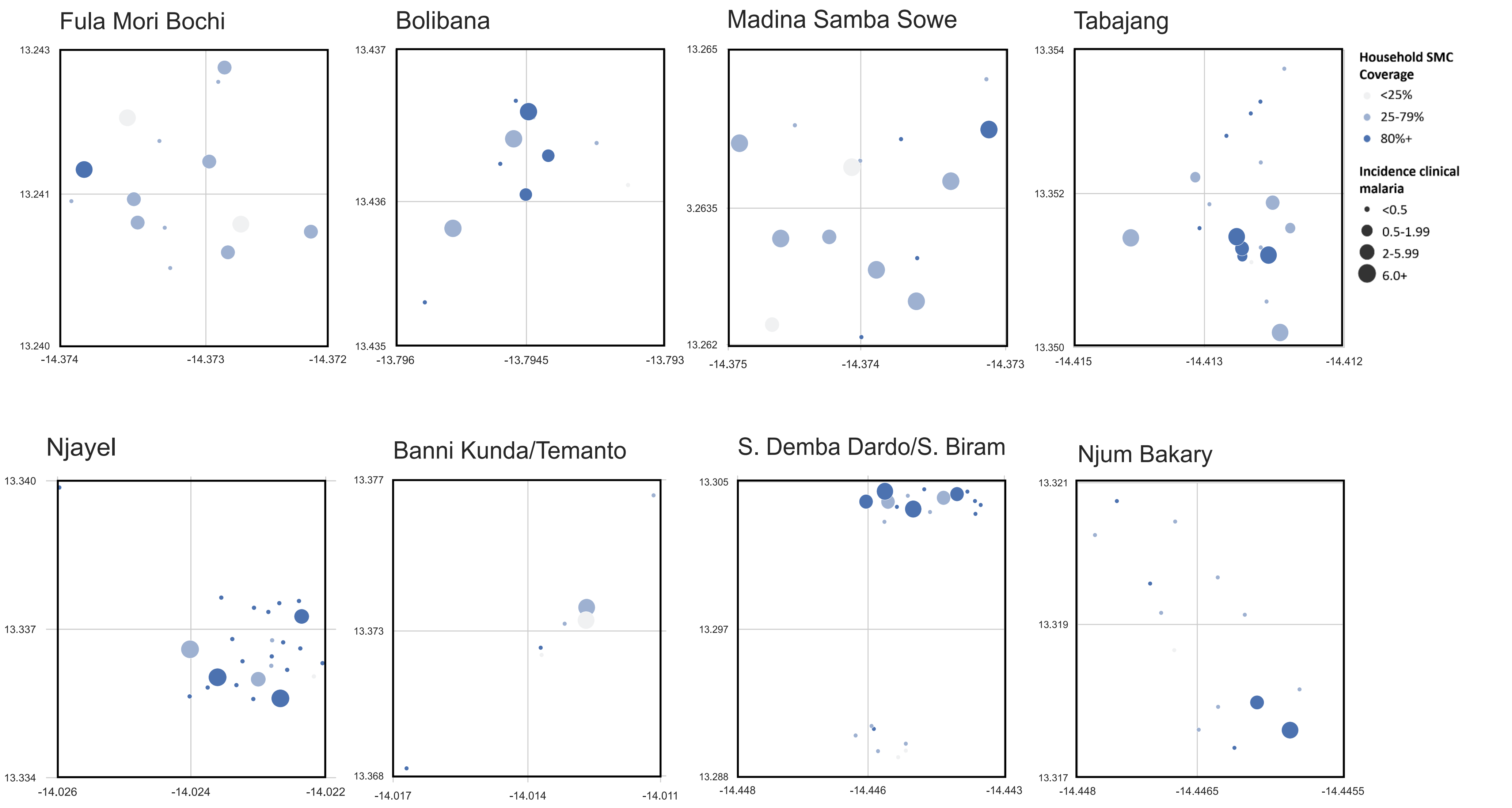

C) **Prevalence of asymptomatic *P. falciparum* infection** detected by qPCR in participants **10+ years of age** from a late seasons survey 27th Sept-27th Dec 2021. By the percentage of children in household who received one or more rounds of SMC.

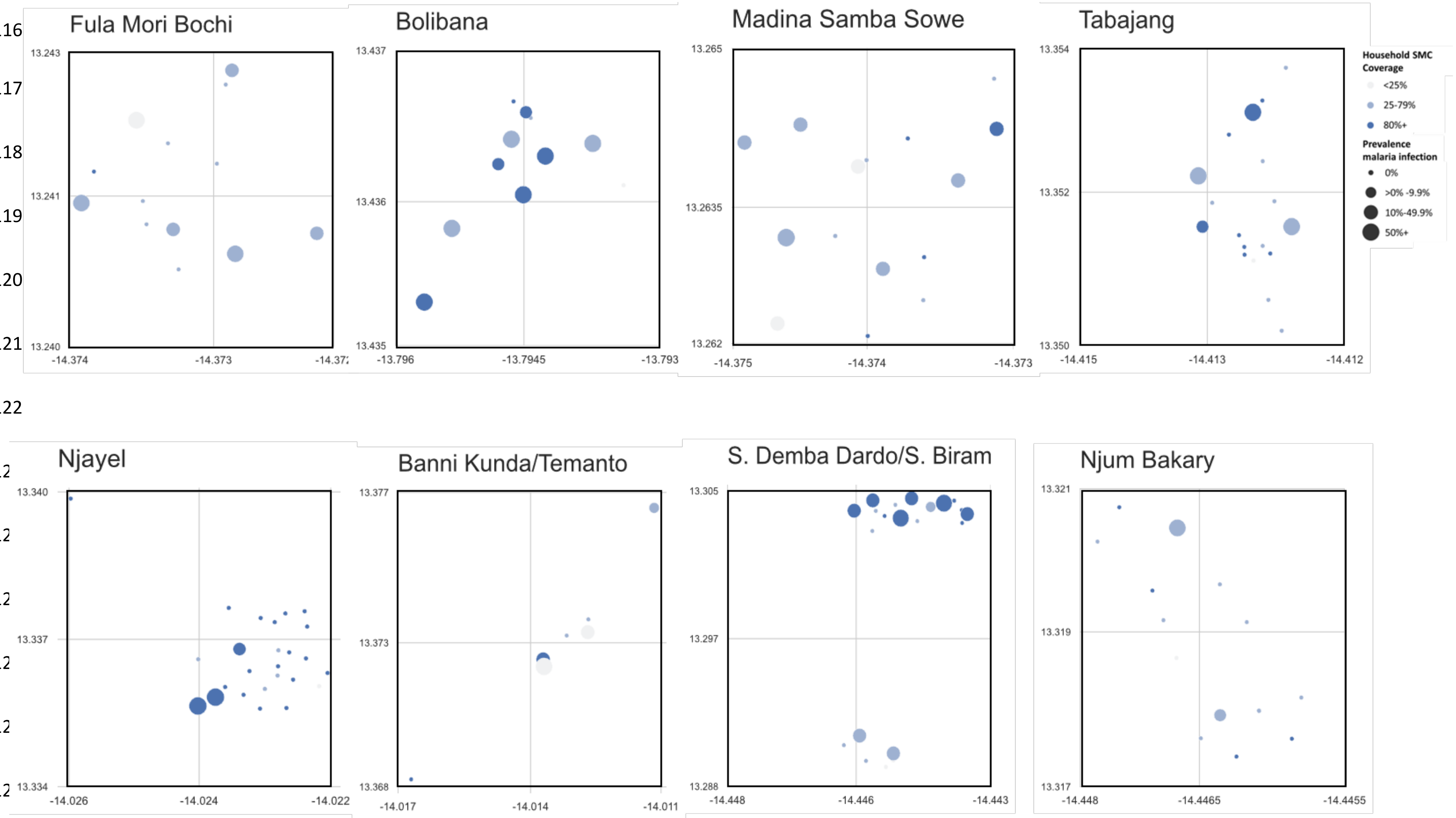

D) **Prevalence of asymptomatic *P. falciparum* infection** in participants **0-9years** detected in qPCR samples from a late seasons survey 27th Sept-27th Dec 2021. By the percentage of children in household who received one or more rounds of SMC

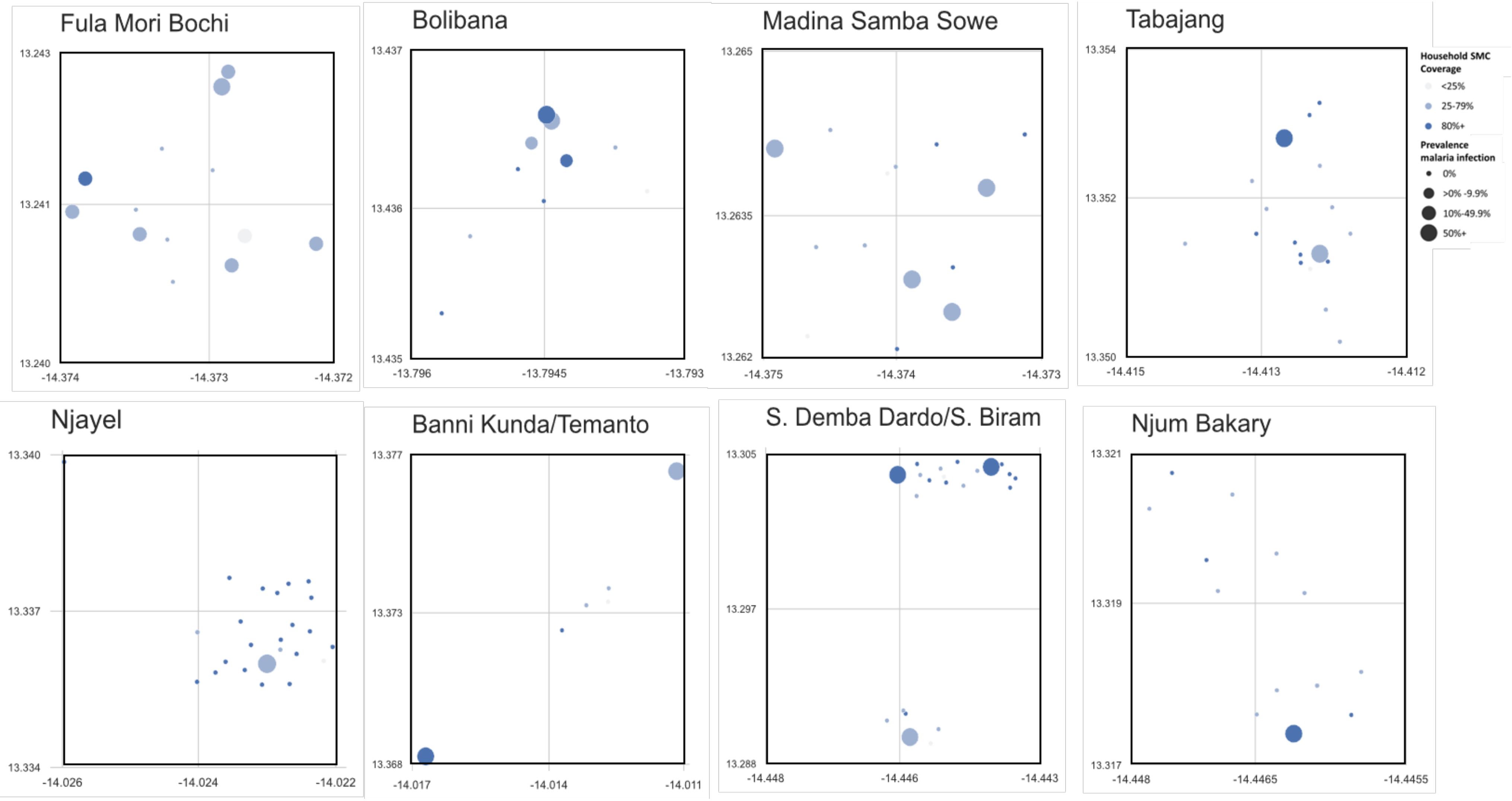

Supplementary Figure 3: P-values from Global Moran I tests for spatial autocorrelation in household-level regression models of malaria incidence and prevalence in participants aged 0-9yrs or 10+yrs by household coverage of SMC.

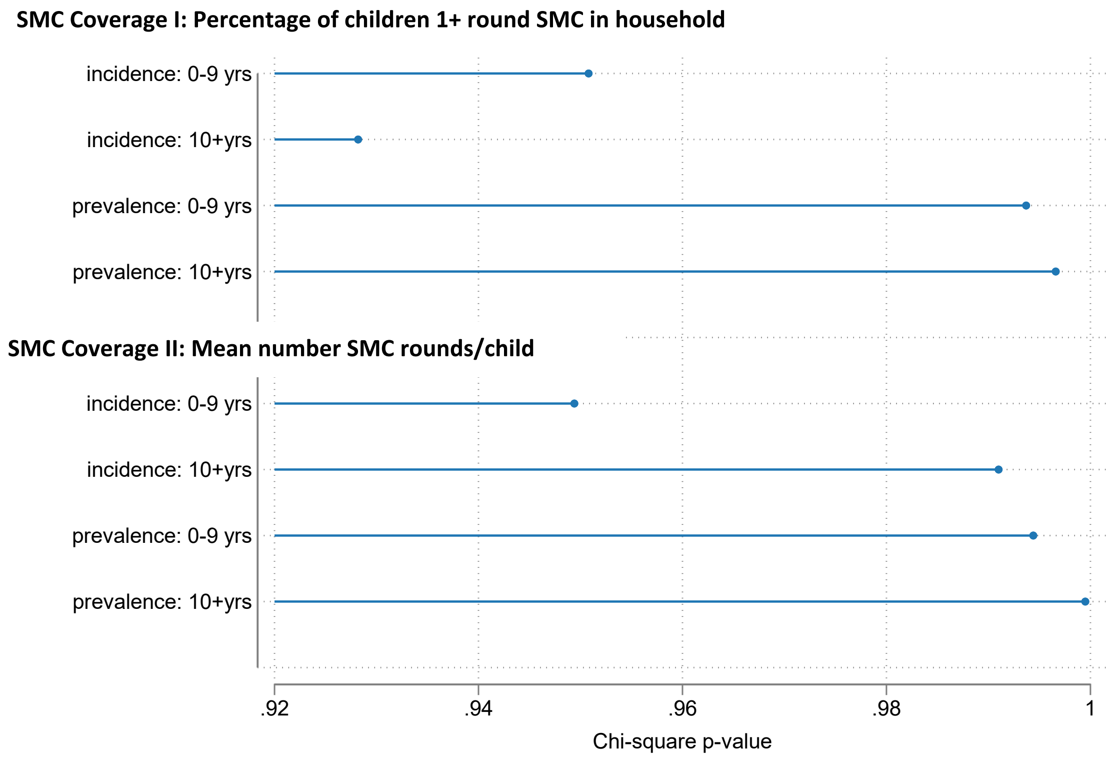

Models regressed household-level malaria outcomes (mean incidence per person in house, or household prevalence at a late season survey 27th Sept-27th Nov 2021) against independent variables measuring household coverage of SMC in eligible children defined in two ways - either the percentage of children in house who received 1+ rounds of SMC (i) or the mean rounds of SMC per eligible child (ii). Models included fixed effects for village ID, ratio of participants 0-9:10+ years, number of instances of reported treated bed net usage the night before out of all instances asked to household members, and the size of household. Post-estimation Moran I tests use the distance matrix for the study site, generated as the inverse weighted distances between households to calculate the probability of spatial autocorrelation in regression errors. P-values above 0.05 indicate the null hypothesis, that there is no spatial autocorrelation, cannot be rejected.
