# Supplementary material for: Household-level effects of seasonal malaria chemoprevention in the Gambia
Source: Commun Med (Lond). 2024 May 22;4:97. doi: 10.1038/s43856-024-00503-0 (PMC11111771; doi:10.1038/s43856-024-00503-0)
Supplement: Supplementary file 3 — Description of Additional Supplementary Files [file 43856_2024_503_MOESM3_ESM.pdf]

Description of Additional Supplementary Files

File name: Supplementary Data 1
Description: Summaries the characteristics of study households by the categories of coverage of children with 1+ round SMC in children aged 0-9 years.

File name: Supplementary Data 2
Description: Summaries the same information by categories of the mean number of SMC rounds per child.

File name: Supplementary Data 3
Description: The source data behind the graphs in the figures.
