# Supplementary material for: Household-level effects of seasonal malaria chemoprevention in the Gambia
Source: Commun Med (Lond). 2024 May 22;4:97. doi: 10.1038/s43856-024-00503-0 (PMC11111771; doi:10.1038/s43856-024-00503-0)
Supplement: Supplementary file 4 — Supplementary Data 1 and 2 [file 43856_2024_503_MOESM4_ESM.pdf]

Supplementary data 1 Characteristics of households by household-level coverage of seasonal malaria chemoprevention in children 0-9 years **(% children at least 1 round SMC)** during the 2021 malaria transmission season, Upper River Region The Gambia.

**Characteristic**	**overall**	**Household level coverage of SMC in children aged 0-9 years**		
		**<25%**	**25% to <80%**	**80%+**
Total households	**129**	**13**	**59**	**57**
Total participants	**2210**	**133**	**1064**	**1013**
% Households with 1+ malaria case over 2021 MTS	86.8% (112)	76.9% (10)	91.5% (54)	84.2% (48)
% Households with children 0-15yrs (% total households)	100.0% (129)	100.0% (13)	100.0% (59)	100.0% (57)
Children aged 0-4yrs	89.9% (116)	69.2% (9)	96.6% (57)	87.7% (50)
Children aged 5-9yrs	96.9% (125)	84.6% (11)	100. 0% (59)	96.5% (55)
Children aged 10-15yrs	93.0% (120)	84.6% (11)	94.9% (56)	92.9% (53)
% Households with participants age 16+ (% total households)	100.0% (127)	100.0% (13)	100. 0% (59)	100.0% (57)
% Households with Female inhabitants	100.0% (129)	100.0% (13)	100.0% (59)	100.0% (57)
% Households with Male inhabitants	100.0% (129)	100.0% (13)	100.0% (59)	100.0% (57)
Average percentage of household inhabitants Fula ethnicity (standard deviation)	48.2 (41.4)	59.5 (37.6)	52.9 (37.9)	40.7 (44.92)
% Long lasting net LLITN coverage in household* (% total households)				
<25%	51.2% (66)	46.1% (6)	52.4% (31)	50.9% (29)
25% - 54%	37.9% (49)	38.5% (5)	37.3% (22)	38.6% (22)
55-79%	7.0% (9)	7.7% (1)	6.8% (4)	7.3% (4)
80%+	3.9% (5)	7.7% (1)	3.4% (2)	3.6% (2)
SMC Coverage				

Household level % coverage (% children in household with 1+ rounds SMC)**				
<25%	10.1% (13)	100.0% (13)	0.0% (0)	0.0% (0)
25-79%	45.7% (59)	0.0% (0)	100.0% (59)	0.0% (0)
80%+	44.2% (57)	0.0% (0)	0.0% (0)	100.0% (57)
Mean number rounds of SMC received per child in household (SD)				
<0.3	10.1% (13)	100.0% (13)	0.0% (0)	0.0% (0)
0.3-2	48.1% (62)	0.0% (0)	84.8% (50)	21.1% (12)
>2	41.9% (54)	0.0% (0)	15.3% (9)	79.0% (45)
Average number persons per household (SD)	17.1 (13.2)	10.2 (9.9)	18.0 (14.3)	17.8 (12.4)
Average number children <10 yrs old per household (SD)	6.2 (5.2)	3.5 (4.2)	6.5 (5.5)	6.4 (4.9)
Average ratio of children to adults per household (0-9 yrs : 10+ yrs) (SD)	0.7 (0.45)	0.7 (0.74)	0.7 (0.50)	0.7 (0.29)
Average number of *An. gambiae* s.l./household/night (SD)	1.4 (1.5)	0.98 (1.0)	1.3 (1.7)	1.6 (1.3)
% households by INDIE intervention arm				
% Control	19.4% (25)	23.1% (3)	26.7% (16)	12.3% (7)
% Fever screen and treat (July-December 2021)	26.4% (34)	23.1% (3)	30.0% (18)	21.1% (12)
%n Mass us-RDT screen and treat (July-December 2021)	24.8% (33)	23.1% (3)	11.7% (7)	38.6% (22)
% Mass drug administration (April-June 2021)	29.5% (38)	30.8% (4)	31.7% (19)	28.1% (63)
Household behaviours – malaria care seeking and treatment (person/household)				
Mean proportion inhabitants with symptoms who sought care	0.39 (0.29)	0.38 (0.39)	0.45 (0.32)	0.34 (0.23)
Mean proportion of visits with reported inhabitant antimalarial use in last 3 weeks	0.06 (0.05)	0.05 (0.06)	0.05 (0.05)	0.07 (0.05)

* Percentage of total visits to each household inhabitant at which inhabitants slept under a long lasting insecticide treated bed net the night before **Three households no SMC data collected during 2021 (MTS)

Supplementary data 2. Characteristics of households by household-level coverage of seasonal malaria chemoprevention in children 0-9 years **(mean number SMC rounds per child**) during the 2021 malaria transmission season, Upper River Region The Gambia

**Characteristic**	**Overall**	**Household level coverage of SMC in children aged 0-9 years – mean rounds per child**		
		**<0.3 rounds/child**	**0.3-2 rounds/child**	**2+ rounds/child**
**Total households**	**129**	**13**	**62**	**54**
**Total participants**	**2120**	**133**	**990**	**1087**
% Households with 1+ malaria case over 2021 MTS	86.8% (112)	76.9% (10)	83.9 (52)	92.6% (50)
% Households with children 0-15yrs (% total households)	100.0% (129)	100.0% (13)	100.0% (62)	100.0% (54)
Children aged 0-4yrs	89.9% (116)	69.2% (9)	88.7% (55)	96.3% (52)
Children aged 5-9yrs	96.9% (125)	84.6% (11)	100.0% (59)	96.3% (52)
Children aged 10-15yrs	93.0% (120)	84.6% (11)	91.9% (57)	96.3% (52)
% Households with participants age 16+ (% total households)	100.0% (129)	100.0% (13)	100.0% (59)	100.0% (56)
% Households with Female inhabitants	100.0% (129)	100.0% (13)	100.0% (59)	100.0% (56)
% Households with Male inhabitants	100.0% (129)	100.0% (13)	98.3% (58)	100.0% (56)
Average percentage of household inhabitants Fula ethnicity (standard deviation)	48.2 (41.4)	59.5 (37.6)	51.0 (39.7)	42.6 (44.0)
**% Long lasting net LLITN coverage in household* (% total households)**				
<25%	51.2% (66)	46.1% (6)	56.5% (35)	43.3% (25)
25% - 54%	38.0% (49)	38.5% (5)	33.9% (21)	42.6% (23)
55-79%	7.0% (9)	7.7% (1)	6.5% (4)	7.4% (4)
80%+	3.9% (5)	7.7% (1)	3.2% (2)	3.7% (2)
**SMC Coverage**				

**Household level % coverage (% children in household with 1+ rounds SMC)****				
<25%	10.1% (13)	100.0% (13)	0.0% (0)	0.0% (0)
25-79%	45.7% (59)	0.0% (0)	80.7% (50)	16.7% (9)
80%+	44.2% (57)	0.0% (0)	19.4% (12)	83.3% (45)
**Household level number rounds of SMC (mean number rounds/child) (SD)**				
<0.3	10.1% (13)	100.0% (13)	0.0% (0)	0.0% (0)
0.3 - <2	48.0% (62)	0.0% (0)	100.0% (13)	0.0% (0)
>2-4	41.9% (54)	0.0% (0)	0.0% (0)	100.0% (54)
Average number persons per household (SD)	17.1 (13.2)	10.2 (9.9)	16.0 (12.8)	20.1 (13.8)
Average number children <10 yrs old per household (SD)	6.2 (5.2)	3.5 (4.2)	5.7 (4.7)	7.4 (5.5)
Average ratio of children to adults per household (0-9 yrs : 10+ yrs) (SD)	0.7 (0.45)	0.7 (0.74)	0.7 (0.50)	0.7 (0.29)
Average number of *An. gambiae* s.l./household/night (SD)	1.4 (1.5)	1.0 (1.0)	1.0 (1.2)	1.9 (1.6)
% households by INDIE intervention arm				
% Control	19.4% (25)	23.1% (3)	27.4% (17)	9.3% (5)
% Fever screen and treat (July-December 2021)	26.4% (34)	23.1% (3)	27.4% (17)	25.9% (14)
%n Mass us-RDT screen and treat (July-December 2021)	24.8% (33)	23.1% (3)	14.5% (9)	37.0% (20)
% Mass drug administration (April-June 2021)	29.5% (38)	30.8% (4)	30.7% (19)	27.8% (15)
Household behaviours – malaria care seeking and treatment (person/household)				
Mean proportion inhabitants with symptoms who sought care	0.39 (0.29)	0.38 (0.39)	0.44 (0.32)	0.34 (0.22)
Mean proportion of visits with reported inhabitant antimalarial use in last 3 weeks	0.06 (0.05)	0.05 (0.06)	0.05 (0.05)	0.06 (0.05)

* Percentage of total visits to each household inhabitant at which inhabitants slept under a long lasting insecticide treated bed net the night before **Three households no SMC data collected during 2021 (MTS)
