# Supplementary material for: Household-level effects of seasonal malaria chemoprevention in the Gambia
Source: Commun Med (Lond). 2024 May 22;4:97. doi: 10.1038/s43856-024-00503-0 (PMC11111771; doi:10.1038/s43856-024-00503-0)
Supplement: Supplementary file 6 — Reporting Summary [file 43856_2024_503_MOESM6_ESM.pdf]

nature portfolio

Corresponding author(s): Seyi Soremekun

Last updated by author(s): Dec 1, 2023

Reporting Summary

Nature Portfolio wishes to improve the reproducibility of the work that we publish. This form provides structure for consistency and transparency in reporting. For further information on Nature Portfolio policies, see our Editorial Policies and the Editorial Policy Checklist.

Statistics

For all statistical analyses, confirm that the following items are present in the figure legend, table legend, main text, or Methods section.

n/a	Confirmed	
☐	☒	The exact sample size (*n*) for each experimental group/condition, given as a discrete number and unit of measurement
☐	☒	A statement on whether measurements were taken from distinct samples or whether the same sample was measured repeatedly
☐	☒	The statistical test(s) used AND whether they are one- or two-sided<br>*Only common tests should be described solely by name; describe more complex techniques in the Methods section.*
☐	☒	A description of all covariates tested
☐	☒	A description of any assumptions or corrections, such as tests of normality and adjustment for multiple comparisons
☐	☒	A full description of the statistical parameters including central tendency (e.g. means) or other basic estimates (e.g. regression coefficient) AND variation (e.g. standard deviation) or associated estimates of uncertainty (e.g. confidence intervals)
☐	☒	For null hypothesis testing, the test statistic (e.g. *F*, *t*, *r*) with confidence intervals, effect sizes, degrees of freedom and *P* value noted<br>*Give P values as exact values whenever suitable.*
☒	☐	For Bayesian analysis, information on the choice of priors and Markov chain Monte Carlo settings
☐	☒	For hierarchical and complex designs, identification of the appropriate level for tests and full reporting of outcomes
☐	☒	Estimates of effect sizes (e.g. Cohen's *d*, Pearson's *r*), indicating how they were calculated

Our web collection on statistics for biologists contains articles on many of the points above.

Software and code

Policy information about availability of computer code

Data collection	Data was collected using Redcap software on handheld tablets and stored on a Redcap server in The Gambia.<br>Copies of the data pertinent to this paper's analysis were transferred to the LSHTM's secure internal servers and converted to Stata .dta datasets.
Data analysis	Data were analysed in Stata v17 and Python v3.9 using software-standard GUI or software-provided code for statistical tests (no custom code or algorithms developed).

For manuscripts utilizing custom algorithms or software that are central to the research but not yet described in published literature, software must be made available to editors and reviewers. We strongly encourage code deposition in a community repository (e.g. GitHub). See the Nature Portfolio guidelines for submitting code & software for further information.

Data

Policy information about availability of data

All manuscripts must include a data availability statement. This statement should provide the following information, where applicable:

- Accession codes, unique identifiers, or web links for publicly available datasets
- A description of any restrictions on data availability
- For clinical datasets or third party data, please ensure that the statement adheres to our policy

Study data is made available via the LSHTM data repository: https://datacompass.lshtm.ac.uk/ and freely accessible upon reasonable request to the repository manager.

Human research participants

Policy information about studies involving human research participants and Sex and Gender in Research.

Reporting on sex and gender	Tables (Table 1 and Supplementary Table 4) reporting the characteristics of the study sample have been disaggregated by sex using the terms 'male' and 'female'.
Population characteristics	The present study included 2210 participants (825 aged 0-9 years and 1,385 participants aged 10-89 years) in 129 households who were met during the 2021 malaria transmission season and had complete address data.
Recruitment	Using a Census list for the study site provided by the Gambia DHS, all inhabitants of 10 communities were invited to take part in the study following community sensitization events in April-May 2021. Full details of the study and outcomes were provided to each household member and participants consented with a signature or thumbprint on a study consent and ethics form. The main caregiver of children under 16 years formally consented on their behalf, in addition to assent given by children aged 12-16 years.
Ethics oversight	Ethical approval was provided by the Government of The Gambia/The MRC Gambia Joint Ethics Committee, The Gambia, and the Ethics Committee of the LSHTM, UK (reference: 16642).

Note that full information on the approval of the study protocol must also be provided in the manuscript.

Field-specific reporting

Please select the one below that is the best fit for your research. If you are not sure, read the appropriate sections before making your selection.

☒ Life sciences ☐ Behavioural & social sciences ☐ Ecological, evolutionary & environmental sciences

For a reference copy of the document with all sections, see nature.com/documents/nr-reporting-summary-flat.pdf

Life sciences study design

All studies must disclose on these points even when the disclosure is negative.

Sample size	This is a secondary analysis using data collected from a trial on malaria transmission dynamics (https://clinicaltrials.gov/ct2/show/NCT04053907). The sample size for this study was determined by the number of participants will full address data from the transmission dynamics study - a sample size calculation at this stage would be tautological.
Data exclusions	Exclusions:<br>404 participants from the study site were never met or did not provide consent.<br>178 Enrolled participants withdrew/were never met during 2021 malaria transmission season<br>39 Enrolled participants had missing household/address information<br>8 Enrolled participants aged <10 years no information on seasonal malaria chemoprevention (SMC) status
Replication	Robustness checks for the main study question:<br>Tests for impact of household SMC coverage on malaria transmission were repeated using two different definitions of household SMC coverage - number of rounds of SMC per eligible child, and the % of eligible children received any SMC.
Randomization	No randomisation was pre-specified: Participants or households in the database were allocated to groups defined by SMC status (individual SMC status of children or or household-level SMC coverage) by the end of the 2021 malaria transmission season in the Upper River Region of The Gambia. This was not random. Analyses comparing outcomes in participants by SMC status were thus adjusted for village ID, insecticide-treated bednet use, the household level prevalence of malaria at a baseline dry season survey in April-May 2021, age in years, the household ratio of children aged 0-9 years to participants aged 10+ years, the total number of household inhabitants, and for prevalence models, the week of survey visit.

Blinding

Blinding is not applicable: this is an observational prospective cohort study of a government programme. The secondary analysis question was devised by the authors after the main study ended, and SMC coverage category definitions informed by the data and conventional cut-offs. During the 2021 malaria transmission season when the routine government SMC programme was active, it would be impossible to blind recipients to their own SMC status (children <10 years) or for older households members, to the approximate coverage of SMC in eligible children.

Reporting for specific materials, systems and methods

We require information from authors about some types of materials, experimental systems and methods used in many studies. Here, indicate whether each material, system or method listed is relevant to your study. If you are not sure if a list item applies to your research, read the appropriate section before selecting a response.

Materials & experimental systems

n/a	Involved in the study
☒	☐ Antibodies
☒	☐ Eukaryotic cell lines
☒	☐ Palaeontology and archaeology
☒	☐ Animals and other organisms
☐	☒ Clinical data
☒	☐ Dual use research of concern

Methods

n/a	Involved in the study
☒	☐ ChIP-seq
☒	☐ Flow cytometry
☒	☐ MRI-based neuroimaging

Clinical data

Policy information about clinical studies

All manuscripts should comply with the ICMJE guidelines for publication of clinical research and a completed CONSORT checklist must be included with all submissions.

Clinical trial registration

The original study from which data used in this paper: https://clinicaltrials.gov/ct2/show/NCT04053907

Study protocol

This study is a secondary analysis of data from the trial registered above, and does not cover any objectives specified in the main trial. The protocol for the original trial is available on request from the Principal Investigator Professor Chris Drakeley Chris.Drakeley@lshtm.ac.uk

Data collection

The setting: Basse District, Upper River Region of the Gambia. The 10 communities included were Njayel, Banni Kunda, Temanto, Bolibana, Fula Mori Bochi, Madina Samba Sowe, Njum Bakary, Sare Demba Dardo, Sare Biram, and Tabajang.
Enrollment occured during a baseline dry season survey in April-May 2021. Data collection occured between April 2021 and January 2022

Outcomes

Incidence of clinical malaria was defined as the number of passively detected cases (care-seeking visits to the community nurse for fever or related symptoms) per 100 person-months. For each clinical case, 2 weeks of follow up were removed from the denominator. Malaria prevalence was defined as the percentage of participants sampled who were qtPCR-positive during a late season survey (27th September to the 27th November 2021). qtPCR-positive participants sampled during this survey were categorised into high- and low-density infections by age group. High-density infections were those with density above the median of the natural log asexual parasite concentrations per µL blood for the age groups (0-4, 5-9, 10-15 and 16+ years).
We used cox survival models to compare incidence between SMC coverage groups, and logistic regression to compare prevalence between SMC coverage groups.
